# Supplementary material for: Effects of Selenium Enrichment on Dough Fermentation Characteristics of Baker’s Yeast
Source: Foods. 2023 Jun 11;12(12):2343. doi: 10.3390/foods12122343 (PMC10297389; doi:10.3390/foods12122343)
Supplement: Supplementary file 1 [file foods-12-02343-s001.zip › foods-2423499-supplementary.pdf]

Table S1 Content and speciation of Se in Se-enriched yeast.

	Total Se content (μg/g)	Inorganic Se content (μg/g)	Organic Se content (μg/g)	SeMet content (μg/g)	SeMet percentage (%)
Original yeast strain	3.77 ±0.58	ND	ND	ND	ND
Se-enriched yeast	3150.56 ± 14.58*	15.29 ± 0.25	3135.27 ± 14.83	2376.89 ± 28.76	75.44 ± 1.28

The asterisk (*) indicated that the experimental group (Se-enriched yeast) had a significant difference compared with the control group (Original yeast strain), $p < 0.05$.
